# Tad pili contribute to the virulence and biofilm formation of virulent *Aeromonas hydrophila*

**DOI:** 10.3389/fcimb.2024.1425624

**Published:** 2024-07-31

**Authors:** Hasan C. Tekedar, Fenny Patel, Jochen Blom, Matt J. Griffin, Geoffrey C. Waldbieser, Salih Kumru, Hossam Abdelhamed, Vandana Dharan, Larry A. Hanson, Mark L. Lawrence

**Affiliations:** ^1^ College of Veterinary Medicine, Mississippi State University, Mississippi State, MS, United States; ^2^ Bioinformatics & Systems Biology, Justus-Liebig-University Giessen, Giessen, Germany; ^3^ Thad Cochran National Warmwater Aquaculture Center, Stoneville, MS, United States; ^4^ Warmwater Aquaculture Research Unit, USDA-ARS, Stoneville, MS, United States; ^5^ Faculty of Fisheries, Recep Tayyip Erdogan University, Rize, Türkiye

**Keywords:** *Aeromonas hydrophila*, virulence, type IV pili, Tad (tight adherence), host-pathogen interaction

## Abstract

Type IV pili (T4P) are versatile proteinaceous protrusions that mediate diverse bacterial processes, including adhesion, motility, and biofilm formation. *Aeromonas hydrophila*, a Gram-negative facultative anaerobe, causes disease in a wide range of hosts. Previously, we reported the presence of a unique Type IV class C pilus, known as tight adherence (Tad), in virulent *Aeromonas hydrophila* (vAh). In the present study, we sought to functionalize the role of Tad pili in the pathogenicity of *A. hydrophila* ML09-119. Through a comprehensive comparative genomics analysis of 170 *A. hydrophila* genomes, the conserved presence of the Tad operon in vAh isolates was confirmed, suggesting its potential contribution to pathogenicity. Herein, the entire Tad operon was knocked out from *A. hydrophila* ML09-119 to elucidate its specific role in *A. hydrophila* virulence. The absence of the Tad operon did not affect growth kinetics but significantly reduced virulence in catfish fingerlings, highlighting the essential role of the Tad operon during infection. Biofilm formation of *A. hydrophila* ML09-119 was significantly decreased in the Tad operon deletant. Absence of the Tad operon had no effect on sensitivity to other environmental stressors, including hydrogen peroxide, osmolarity, alkalinity, and temperature; however, it was more sensitive to low pH conditions. Scanning electron microscopy revealed that the Tad mutant had a rougher surface structure during log phase growth than the wildtype strain, indicating the absence of Tad impacts the outer surface of vAh during cell division, of which the biological consequences are unknown. These findings highlight the role of Tad in vAh pathogenesis and biofilm formation, signifying the importance of T4P in bacterial infections.

## 1 Introduction

Aquaculture helps meet the ever-growing global demand for animal protein, yet it faces a substantial threat from fish pathogens, leading to economic losses and food insecurity (Mzula et al., 2019; Irshath et al., 2023). Among these pathogens, several *Aeromonas* species are prominent agents of Motile Aeromonas Septicemia (MAS) in temperate and warm freshwater fish, with *Aeromonas hydrophila* being a significant contributor (Irshath et al., 2023).

*A. hydrophila* is a Gram-negative facultative anaerobe that thrives in fresh and brackish water environments. It possesses a wide host range which includes amphibians, fish, birds, reptiles, and mammals, including humans (Huys et al., 2003; Tsai et al., 2015; Laviad-Shitrit et al., 2018), causing septicemia, necrotizing fasciitis, and gastroenteritis (Parker and Shaw, 2011; Tomás, 2012; Tsai et al., 2015; Pessoa et al., 2022). Consequently, extensive research has centered on investigating the underlying virulence mechanisms of *A. hydrophila*. Multiple virulence factors have been identified, including iron-binding systems, flagella, adhesins, exotoxins, enterotoxins, secretion systems, S-layers, and fimbriae (Horneman et al., 2009; Tomás, 2012; Rasmussen-Ivey et al., 2016a; Davidsson et al., 2017; Barger et al., 2020). A particular clonal type of *A. hydrophila* affiliated with sequence type 251 (ST251) is highly virulent in catfish and carp species in aquaculture, referred hereafter as virulent *A. hydrophila* (vAh) (Rasmussen-Ivey et al., 2016b).

Fimbriae (pili) are long extracellular polymers that enable bacteria to interact with their external environment (Hospenthal et al., 2017). Type IV pili (T4P) are widely distributed across bacterial and archaeal phyla and allow for twitching motility, DNA uptake through natural transformation, host colonization and signaling, chemotaxis, surface sensing, and virulence protein secretion (Cehovin et al., 2013; Angelov et al., 2015; Kåhrström, 2015; Piepenbrink, 2019; Mix et al., 2021; Oliveira et al., 2022). T4P, spanning both the outer and inner membrane, are divided into two subclasses, type IV class a pili (T4aP) and type IV class b pili (T4bP), based on the length of their prepilin leader sequence (Pelicic, 2008; McCallum et al., 2019; Ellison et al., 2022).

A third subclass of T4P, Type IV class c pili (T4cP), was recently classified due to the distinct genetic organization and evolutionary history of Tight Adherence Pili (Tad) (Beeby, 2019; Denise et al., 2019). Tad pili, originally discovered in *Actinobacillus actinomycetemcomitans*, are crucial for forming bundled fibers that facilitate autoaggregation, adherence, colonization, and biofilm development (Kachlany et al., 2000). It has been postulated that Tad originated via horizontal acquisition from Archaea given their significant similarity to archaeal Epd-like systems (Denise et al., 2019). For example, Tad, located on single locus, lacks a retraction ATPase and instead encodes a bifunctional ATPase responsible for both extension and retraction processes (Beeby, 2019; Ellison et al., 2019; Piepenbrink, 2019).

*A. hydrophila* exhibits outer membrane-spanning type I pili and T4P. *A. hydrophila* encodes two distinct types of T4aP: Mannose-Sensitive Hemagglutinin (MSHA) and Type IV Aeromonas Pilus (Tap). MSHA bundle forming pili shares high homology to the MSHA pili of *Vibrio* spp., while Tap pili shares high homology to the Pil system of *Vibrio* and *Pseudomonas* spp. In addition, previous research reported the consistent presence of genes encoding Tad pili in vAh genomes (Tekedar et al., 2019). Notably, the Tad cluster was also found in a clinical *A. hydrophila* isolate infecting humans, suggesting potential significance in pathogenicity (Tekedar et al., 2019). However, within the context of vAh pathogenicity, the precise functions of the T4P have not been investigated previously.

The Tad system, with its diverse functions, serves as a bridge that facilitates interaction between the pathogen and its host during infection. This study aimed to delineate the function of Tad pili in vAh isolate ML09-119. We conducted an expanded comparative genomics analysis involving 170 *A. hydrophila* genomes sourced from the National Center for Biotechnology Information (NCBI), confirming the consistent presence of the Tad gene cluster in vAh genomes. We constructed a mutant strain with complete deletion of the Tad operon and investigated its contribution to virulence in catfish. Additionally, the influence of Tad pili on *A. hydrophila* physiology and response to environmental stress was determined. These results revealed the significant role of Tad pili in the virulence of vAh isolate ML09-119, highlighting its crucial function in mediating pathogenic interactions.

## 2 Materials and methods

### 2.1 Genome features and building phylogenetic tree

Genome sequence data for 170 *A. hydrophila* genomes were downloaded from the NCBI database. Genome metadata is listed in **Table 1**. All selected genomes shared at least 95% average nucleotide identity (ANI) among them, indicating conspecificity, and a phylogenetic tree was built from the core genome (Blom et al., 2016). Also, AAI was calculated with EDGAR 2.0 (Blom et al., 2016). Gene sets from the core genome were aligned individually using MUSCLE (Edgar, 2004) then concatenated. The concatenated alignment was used to compute a Kimura distance matrix, which was served as input for the Neighbor-Joining algorithm implemented in PHYLP (Baum, 1989). The phylogenetic tree for 170 genomes was build out of a core of 1893 genes per genome, 321810 in total. The core has 1711702 AA-residues/bp per genome, 290989340 in total.

**Table 1:** Genome features for 170 *A. hydrophila* genomes utilized in this study.

#	Species	Strain	Location	Source	Level	Accession	References
**1**	*A. hydrophila*	LZ-MG14	China	Activated sludge	Contig	NZ_JAANAW010000000	(Ji et al., 2020)
**2**	*A. hydrophila*	S5	USA	Wastewater	Scaffold	JARESK000000000	N\A
**3**	*A. hydrophila*	ZYAH75	China	Clinical	Complete	NZ_CP016990	(Islam et al., 2019)
**4**	*A. hydrophila*	CL1	Japan	Hospital sewage	Contig	JARADF000000000	(Ota et al., 2023)
**5**	*A. hydrophila*	Aer_On19M	Brazil	Tilapia	Scaffold	NZ_JAADJL010000001	N\A
**6**	*A. hydrophila*	Aer_On29M	Brazil	Tilapia	Scaffold	NZ_WSYM01000001	N\A
**7**	*A. hydrophila*	NUITM-VA1	Vietnam	NA	Complete	NZ_AP025277	(Dao et al., 2022)
**8**	*A. hydrophila*	LaG38	Brazil	*Lophiosilurus alexandri* (Catfish)	Contig	NZ_WOCB01000000	N\A
**9**	*A. hydrophila*	Aer_OnAA	Brazil	Tilapia	Scaffold	NZ_WTZB01000001	N\A
**10**	*A. hydrophila*	Brac54	Brazil	*Dendrocephalus brasiliensis* (shrimp)	Scaffold	NZ_JAABKF010000000	N\A
**11**	*A. hydrophila*	S-P-C-022.01	Sudan	*Homo sapiens*	Complete	CP092709	N\A
**12**	*A. hydrophila*	Aer_Brac66	Brazil	*Dendrocephalus brasiliensis* (shrimp)	Complete	NZ_CP045220	N\A
**13**	*A. hydrophila*	Aer_LaG33	Brazil	*Lophiosilurus alexandri* (catfish)	Complete	NZ_CP046871	N\A
**14**	*A. hydrophila*	Brac6	Brazil	*Dendrocephalus brasiliensis* (shrimp)	Complete	NZ_CP050850	N\A
**15**	*A. hydrophila*	Aer_OnP4.2	Brazil	Tilapia	Complete	NZ_CP046870	N\A
**16**	*A. hydrophila*	OnP3.1	Brazil	Tilapia	Complete	NZ_CP050851	N\A
**17**	*A. hydrophila*	Aer_Brac14A	Brazil	*Dendrocephalus brasiliensis* (shrimp)	Complete	NZ_CP045502	N\A
**18**	*A. hydrophila*	Aer_OnP2.2	Brazil	Tilapia	Complete	NZ_CP046869	N\A
**19**	*A. hydrophila*	Aer_LaG34	Brazil	*Lophiosilurus alexandri* (catfish)	Complete	NZ_CP046604	N\A
**20**	*A. hydrophila*	CN17A0055	China	*Homo sapiens*	Contig	NZ_JAEHHS010000000	(Du et al., 2021)
**21**	*A. hydrophila*	CN17A0062	China	*Homo sapiens*	Contig	NZ_JAEHHP010000000	(Du et al., 2021)
**22**	*A. hydrophila*	M013	Malaysia	Waterfall	Contig	NZ_JRWS00000000.1	(Tan et al., 2015a)
**23**	*A. hydrophila*	S00023	USA	*Heterelmis comalensis*	Scaffold	JANUCE000000000	N\A
**24**	*A. hydrophila*	S00040	USA	*Heterelmis comalensis*	Scaffold	JANUCG000000000	N\A
**25**	*A. hydrophila*	KAM461	Japan	NA	Contig	NZ_BQVF01000000	N\A
**26**	*A. hydrophila*	MGYG-HGUT-02526	USA	Human gut	Contig	NZ_CABMNZ010000000 N\A	
**27**	*A. hydrophila*	AHNIH1	USA	Clinical	Complete	NZ_CP016380.1	(Hughes et al., 2016)
**28**	*A. hydrophila*	AL06-06	USA	Goldfish	Complete	NZ_CP010947.1	(Tekedar et al., 2015)
**29**	*A. hydrophila*	71328	China	*Homo sapiens*	Contig	NZ_JAJDSS010000001	N\A
**30**	*A. hydrophila*	A058	China	Snakehead fish	Scaffold	JANADJ000000000	N\A
**31**	*A. hydrophila*	PAQ091014-12	USA	Trout	Scaffold	NZ_NKWC01000000	(Rangel et al., 2019)
**32**	*A. hydrophila*	TN-97-08	USA	Bluegill	Contig	NZ_LNUR00000000.1	(Tekedar et al., 2016a)
**33**	*A. hydrophila*	AH10	China	Grass carp	Complete	NZ_CP011100.1	(Xu et al., 2013)
**34**	*A. hydrophila*	AC185	South Korea	Eel	Complete	NZ_CP093308	(Ho et al., 2023)
**35**	*A. hydrophila*	Aer_On27M	Brazil	Tilapia	Scaffold	NZ_WTZJ01000001	N\A
**36**	*A. hydrophila*	SRR12456162	United Kingdom	Fish gut metagenome	Contig	CAMFKU000000000	N\A
**37**	*A. hydrophila*	SRR9109406	United Kingdom	Fish gut metagenome	Contig	CAMFIN000000000	N\A
**38**	*A. hydrophila*	117	China	Goldfish	Scaffold	JANADI010000000	N\A
**39**	*A. hydrophila*	53_AHYD	USA	Clinical	Scaffold	NZ_JVDL00000000.1	(Roach et al., 2015)
**40**	*A. hydrophila*	56_AHYD	USA	Clinical	Scaffold	NZ_JVCD00000000.1	(Roach et al., 2015)
**41**	*A. hydrophila*	48_AHYD	USA	Clinical	Scaffold	NZ_JVFM00000000.1	(Roach et al., 2015)
**42**	*A. hydrophila*	52_AHYD	USA	Clinical	Scaffold	NZ_JVDW00000000.1	(Roach et al., 2015)
**43**	*A. hydrophila*	AH1	USA	Clinical	Scaffold	LSZC00000000	N\A
**44**	*A. hydrophila*	A-1	USA	*Homo sapiens*	Contig	NZ_JACLAO010000001	(Hilt et al., 2020)
**45**	*A. hydrophila*	B-2	USA	*Homo sapiens*	Contig	NZ_JACLAM010000000	(Hilt et al., 2020)
**46**	*A. hydrophila*	AFG_SD03_1510_Ahy_093	Afghanistan	Dog	Contig	NZ_PUTQ00000000	(Boehmer et al., 2018)
**47**	*A. hydrophila*	HIa5mS2	Japan	Hospital sewage	Contig	NZ_JAFIMN010000000	N\A
**48**	*A. hydrophila*	WP7-S18-ESBL-06	Japan	Wastewater	Complete	NZ_AP022206	(Sekizuka et al., 2022)
**49**	*A. hydrophila*	A008N2	China	Water	Complete	NZ_CP094267	N\A
**50**	*A. hydrophila*	MX16A	China	Water	Complete	NZ_CP018201	(Guo et al., 2022)
**51**	*A. hydrophila*	71339	China	*Homo sapiens*	Complete	NZ_CP084352	N\A
**52**	*A. hydrophila*	Ah2101	China	Clinical	Scaffold	NZ_JALKAG010000000	(Xu et al., 2022)
**53**	*A. hydrophila*	Ah2111	China	*Homo sapiens*	Complete	NZ_CP095280.1	(Xu et al., 2022)
**54**	*A. hydrophila*	34SFC-3	Brazil	Seawater	Scaffold	JAPESZ000000000	(Canellas et al., 2023)
**55**	*A. hydrophila*	CSUSB2	USA	American alligator’s water tank	Complete	NZ_CP083944	(Sanders et al., 2022)
**56**	*A. hydrophila*	BT-2012-871	Vietnam	Catfish	Contig	JALRNL000000000	(Ngo et al., 2022)
**57**	*A. hydrophila*	LP0103	Taiwan	*Hypostomus plecostomus*	Complete	NZ_CP092906	(Bureros et al., 2022)
**58**	*A. hydrophila*	SRR3330157	United Kingdom	Fish gut metagenome	Contig	CALCDU000000000	N\A
**59**	*A. hydrophila*	GSH8-2	Japan	Wastewater	Complete	NZ_AP019193	(Sekizuka et al., 2019)
**60**	*A. hydrophila*	WP8-S18-ESBL-02	Japan	Wastewater	Complete	NZ_AP022252	(Sekizuka et al., 2022)
**61**	*A. hydrophila*	NF2	USA	Clinical	Contig	NZ_JDWC00000000.1	(Grim et al., 2014)
**62**	*A. hydrophila*	Aer_WatGTCBM23	Brazil	Tilapia	Scaffold	NZ_WSYK01000001	N\A
**63**	*A. hydrophila*	GTCBM_22	Brazil	Water	Scaffold	NZ_JAACMX010000000	N\A
**64**	*A. hydrophila*	2014-10509-28-27	USA	Fish	Scaffold	NZ_NKXI01000017	(Rangel et al., 2019)
**65**	*A. hydrophila*	Aer_On3M	Brazil	Tilapia	Scaffold	NZ_WTZD01000001	N\A
**66**	*A. hydrophila*	Aer_OnRU2	Brazil	Tilapia	Scaffold	NZ_WTZC01000001	N\A
**67**	*A. hydrophila*	Aer_OnRU3	Brazil	Tilapia	Scaffold	NZ_WTZE01000001	N\A
**68**	*A. hydrophila*	AL97-91	USA	Tilapia	Contig	NZ_CM004591.1	(Tekedar et al., 2017)
**69**	*A. hydrophila*	MN98-04	USA	Tilapia	Contig	NZ_CM004592.1	(Tekedar et al., 2017)
**70**	*A. hydrophila*	T4	Bangladesh	*Labeo rohita* (carp)	Complete	NZ_LR963141	N\A
**71**	*A. hydrophila*	BAQ071013-136	USA	Perch	Scaffold	NZ_NKWV01000000	(Rangel et al., 2019)
**72**	*A. hydrophila*	RB-AH	Malaysia	Soil	Contig	NZ_JPEH00000000.1	(Emond-Rheault et al., 2015)
**73**	*A. hydrophila*	AH-1	Canada	Moribund fish	Contig	NZ_LYXN00000000.1	(Forn-Cuní et al., 2016)
**74**	*A. hydrophila*	KAM385	Japan	NA	Contig	NZ_BPOS01000000	N\A
**75**	*A. hydrophila*	WCHAH045096	China	Sewage	Complete	NZ_CP028568	N\A
**76**	*A. hydrophila*	PAQ091014-21	USA	Trout	Scaffold	NZ_NKWA01000000	(Rangel et al., 2019)
**77**	*A. hydrophila*	ARS-131-14	USA	Fish	Scaffold	NZ_NKXB01000000	(Rangel et al., 2019)
**78**	*A. hydrophila*	PAQ091014-1	USA	Trout	Scaffold	NZ_NKWF01000000	(Rangel et al., 2019)
**79**	*A. hydrophila*	PAQ091014-9	USA	Trout	Scaffold	NZ_NKWD01000000	(Rangel et al., 2019)
**80**	*A. hydrophila*	Aer_On1M	Brazil	Tilapia	Scaffold	NZ_JAADJN010000001	N\A
**81**	*A. hydrophila*	RIMD111065	Japan	*Homo sapiens*	Complete	NZ_AP024234	(Yamazaki et al., 2020)
**82**	*A. hydrophila*	On9M	Brazil	Tilapia	Contig	NZ_WNLG01000000	N\A
**83**	*A. hydrophila*	Ah_On10M	Brazil	Tilapia	Scaffold	NZ_JACSWK010000000	N\A
**84**	*A. hydrophila*	Ah_On23M	Brazil	Tilapia	Scaffold	NZ_JACSWL010000000	N\A
**85**	*A. hydrophila*	Aer_On22M	Brazil	Tilapia	Scaffold	NZ_WTZF01000001	N\A
**86**	*A. hydrophila*	Ah_On16M	Brazil	Tilapia	Scaffold	NZ_JACSWM010000000	N\A
**87**	*A. hydrophila*	RIT668	USA	*Clemmys guttata* (spotted turtle)	Contig	NZ_JABAJN010000000	(Thomas et al., 2020)
**88**	*A. hydrophila*	VL-2012-870	Vietnam	Catfish	Contig	JALRNK000000000	(Xu et al., 2023)
**89**	*A. hydrophila*	ATCC 7966	USA	Milk tin	Contig	NZ_JAGDEM010000000	(Seshadri et al., 2006)
**90**	*A. hydrophila*	NCTC8049	NA	NA	Contig	NZ_UFSL01000000	(Seshadri et al., 2006)
**91**	*A. hydrophila*	ATCC 7966	USA	Milk tin	Complete	NC_008570.1	(Seshadri et al., 2006)
**92**	*A. hydrophila*	Aer_Brac46	Brazil	*Dendrocephalus brasiliensis* (shrimp)	Scaffold	NZ_JAABKI010000001	N\A
**93**	*A. hydrophila*	Aer_Brac14	Brazil	Tilapia	Scaffold	NZ_WTZI01000001	N\A
**94**	*A. hydrophila*	Pi11	Brazil	*Phractocephalus hemioliopterus*	Scaffold	NZ_JAABKG010000000	N\A
**95**	*A. hydrophila*	CN17A0136	China	*Homo sapiens*	Contig	NZ_JAEHIT010000000	(Du et al., 2021)
**96**	*A. hydrophila*	NF1	USA	Clinical	Contig	NZ_JDWB00000000.1	N\A
**97**	*A. hydrophila*	3019	USA	Fish	Complete	CP053885.1	(Wang et al., 2021)
**98**	*A. hydrophila*	AC133	South Korea	Carp	Complete	NZ_CP093309	N\A
**99**	*A. hydrophila*	BSK-10	China	Crucian carp	Scaffold	NZ_NBOV00000000	N\A
**100**	*A. hydrophila*	NJ-35	China	Carp	Complete	NZ_CP006870.1	(Pang et al., 2015)
**101**	*A. hydrophila*	GYK1	China	*Siniperca chuatsi*	Complete	NZ_CP016392.1	(Pan H et al., 2004)
**102**	*A. hydrophila*	DT-TTD-2020-734	Vietnam	Catfish	Contig	JALRNI000000000	(Xu et al., 2023)
**103**	*A. hydrophila*	vAh ST251	Vietnam	*Pangasianodon hypophthalmus*	Complete	NZ_LR963135	(Pang et al., 2015)
**104**	*A. hydrophila*	J-1	China	Carp	Complete	NZ_CP006883.1	(Pang et al., 2015)
**105**	*A. hydrophila*	HZAUAH	China	Crucian carp	Scaffold	NZ_MRDF00000000	(Teng et al., 2017)
**106**	*A. hydrophila*	Ah DBHS101	China	Bighead carp	Scaffold	NZ_NADM00000000	(Xu-Jie et al., 2013)
**107**	*A. hydrophila*	W37	China	Fish	Scaffold	NZ_QQBF01000000	N\A
**108**	*A. hydrophila*	ZYAH72	China	Crucian carp	Complete	NZ_CP016989	(Li et al., 2021)
**109**	*A. hydrophila*	LPL-1	China	Sturgeon	Contig	NZ_JAIWZB010000000	(Wang et al., 2022)
**110**	*A. hydrophila*	AL09-79	USA	Catfish	Contig	NZ_LRRV00000000.1	(Tekedar et al., 2016b)
**111**	*A. hydrophila*	ML09-121	USA	Catfish	Contig	NZ_LRRX00000000.1	(Tekedar et al., 2016b)
**112**	*A. hydrophila*	AL10-121	USA	Catfish	Contig	NZ_LRRW00000000.1	(Tekedar et al., 2016b)
**113**	*A. hydrophila*	ML09-122	USA	Catfish	Contig	NZ_LRRY00000000.1	(Tekedar et al., 2016b)
**114**	*A. hydrophila*	Arkansas 2010	USA	Catfish	Contig	NZ_LYZH00000000.1	(Tekedar et al., 2017)
**115**	*A. hydrophila*	pc104A	USA	Soil	Complete	NZ_CP007576.1	(Pridgeon et al., 2014a)
**116**	*A. hydrophila*	ML09-119	USA	Catfish	Complete	NC_021290.1	(Tekedar et al., 2013)
**117**	*A. hydrophila*	AL09-71	USA	Catfish	Complete	NZ_CP007566.1	(Pridgeon et al., 2014b)
**118**	*A. hydrophila*	Ah27	China	Catfish	Complete	NZ_CP084581	(Li et al., 2023)
**119**	*A. hydrophila*	4LNC202	China	Silver carp	Scaffold	NZ_MJGE01000001	N\A
**120**	*A. hydrophila*	4LNG101	China	Silver carp	Scaffold	MJGY00000000	N\A
**121**	*A. hydrophila*	D4	China	*Megalobrama amblycephala*	Complete	NZ_CP013965.1	(Zhu et al., 2019)
**122**	*A. hydrophila*	LHW39	China	*Megalobrama amblycephala*	Complete	NZ_CP050012	N\A
**123**	*A. hydrophila*	2JBN101	China	Crucian carp	Contig	NZ_LXME00000000.1	(Xu-Jie et al., 2013)
**124**	*A. hydrophila*	JBN2301	China	Carp	Complete	NZ_CP013178.1	(Yang et al., 2016)
**125**	*A. hydrophila*	CN17A0078	China	*Homo sapiens*	Contig	NZ_JAEHJS010000000	(Du et al., 2021)
**126**	*A. hydrophila*	VL-2013-869	Vietnam	Catfish	Contig	JALRNJ000000000	(Xu et al., 2023)
**127**	*A. hydrophila*	71317	China	*Homo sapiens*	Complete	NZ_CP084353	N\A
**128**	*A. hydrophila*	Aer_Pi25.1HTAS	Brazil	*Phractocephalus hemioliopterus* (redtail catfish)	Complete	NZ_CP045501	N\A
**129**	*A. hydrophila*	2961	USA	Chicken	Contig	VHIX01000000	(Wang et al., 2021)
**130**	*A. hydrophila*	RU34A	USA	NA	Scaffold	NZ_FTME00000000	N\A
**131**	*A. hydrophila*	RU34C	USA	NA	Scaffold	NZ_FTNG01000000	N\A
**132**	*A. hydrophila*	CN17A0135	China	*Homo sapiens*	Contig	NZ_JAEHIU010000000	(Du et al., 2021)
**133**	*A. hydrophila*	PW01	China	Pool water	Scaffold	NZ_LZDC01000000	N\A
**134**	*A. hydrophila*	A34a	South Africa	Pig	Contig	NZ_VWTU01000001	N\A
**135**	*A. hydrophila*	SD/21-01	India	*Labeo rohita*	Contig	JAJVCT000000000	(Dubey et al., 2022)
**136**	*A. hydrophila*	Ae25	Sri Lanka	Carp	Contig	BEYT01000045	(Honein et al., 2018)
**137**	*A. hydrophila*	S73-1	USA	Organic broccoli slaw	Contig	NZ_JAFLWS010000000	(Moon et al., 2021)
**138**	*A. hydrophila*	SNUFPC-A8	South Korea	Salmon	Contig	NZ_AMQA00000000.1	(Han et al., 2013)
**139**	*A. hydrophila*	CN17A0134	China	*Homo sapiens*	Contig	NZ_JAEHIV010000000	(Du et al., 2021)
**140**	*A. hydrophila*	PSKL.DP	Hong Kong	Stormwater drain	Complete	CP117918	N\A
**141**	*A. hydrophila*	S0629	USA	*Heterelmis comalensis*	Contig	JAMZGE000000000	N\A
**142**	*A. hydrophila*	S0637	USA	*Heterelmis comalensis*	Contig	JAMZGL000000000	N\A
**143**	*A. hydrophila*	S541	China	River water	Contig	JANKLR000000000	(Xu et al., 2022)
**144**	*A. hydrophila*	TK181	Nigeria	Water	Contig	NZ_JAIOGR010000000	N\A
**145**	*A. hydrophila*	KAM330	Japan	NA	Complete	NZ_AP023398	N\A
**146**	*A. hydrophila*	AS12	Japan	Hospital sewage	Contig	JARADG000000000	(Ota et al., 2023)
**147**	*A. hydrophila*	K522	China	*Homo sapiens*	Complete	CP118699	N\A
**148**	*A. hydrophila*	Ah-HSP	Brazil	Clinical	Contig	NZ_CM007660	(Moura et al., 2017)
**149**	*A. hydrophila*	USC WW 2022	USA	Influent wastewater	Scaffold	JAOWMC000000000	(Wang and Smith, 2023)
**150**	*A. hydrophila*	516	China	Aquatic animal	Complete	CP117470.1	N\A
**151**	*A. hydrophila*	SD/21-05	India	*Labeo rohita*	Contig	JAJVCU000000000	(Dubey et al., 2022)
**152**	*A. hydrophila*	23-C-23	China	NA	Complete	NZ_CP038465	(Liu et al., 2021)
**153**	*A. hydrophila*	WCX23	China	Snake	Complete	NZ_CP038463	(Liu et al., 2019)
**154**	*A. hydrophila*	YURI 17	Taiwan	*Homo sapiens*	Complete	CP121796	N\A
**155**	*A. hydrophila*	M023	Malaysia	Waterfall	Contig	NZ_JSWA00000000.1	(Tan et al., 2015b)
**156**	*A. hydrophila*	AD9	USA	Wetland sediment	Contig	NZ_JFJO00000000.1	(Lenneman and Barney, 2014)
**157**	*A. hydrophila*	TPS-30	China	Fish	Scaffold	NZ_NBWY00000000	N\A
**158**	*A. hydrophila*	G21616-S1	Germany	*Nematostella vectensis*	Scaffold	NZ_JAPWHH010000000	N\A
**159**	*A. hydrophila*	4960	USA	Chicken	Complete	CP053883	(Wang et al., 2021)
**160**	*A. hydrophila*	M894	China	River water	Contig	JANKLS000000000	N\A
**161**	*A. hydrophila*	Aer284	Brazil	Clinical	Scaffold	NZ_RQKC00000000	(de Melo et al., 2019)
**162**	*A. hydrophila*	KN-Mc-1R2	South Korea	*Myocastor coypus*	Complete	NZ_CP027804	(Lim et al., 2020)
**163**	*A. hydrophila*	HX-3	China	*Larimichthys crocea*	Complete	NZ_CP046954	(Jin et al., 2020)
**164**	*A. hydrophila*	Ae34	Sri Lanka	Carp	Contig	NZ_BAXY00000000.1	(Jagoda et al., 2014)
**165**	*A. hydrophila*	FDAARGOS_916	USA	NA	Complete	NZ_CP065651	N\A
**166**	*A. hydrophila*	RM8376	NA	NA	Complete	NZ_CP064382	N\A
**167**	*A. hydrophila*	M062	Malaysia	Waterfall	Contig	NZ_JSXE00000000.1	(Chan et al., 2015)
**168**	*A. hydrophila*	M052	Malaysia	Waterfall	Contig	NZ_MAKI00000000.1	N\A
**169**	*A. hydrophila*	M053	Malaysia	Waterfall	Contig	NZ_MAKJ00000000.1	N\A
**170**	*A. hydrophila*	AHNIH2	USA	Environment	Contig	NZ_PQLL00000000	N\A

### 2.2 Distribution of individual Tad operon genes in 170 *A. hydrophila* genomes

**Figure 1:**
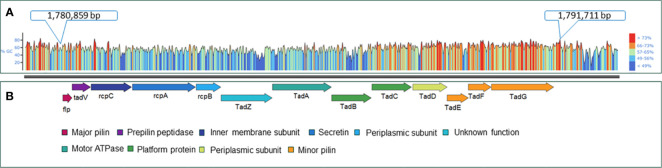
Genetic map of the *A*. *hydrophila* ML09-119 Tad pilus operon and its component genes. **(A)** GC content analysis of the Tad operon within the *A*. *hydrophila* ML09-119 genome. The genome location of the Tad operon is indicated in the figure. **(B)** The components of the Tad operon, along with their predicted or known functions, are indicated. The Tad genes are color-coded based on their respective functions.

**Figure 2:**
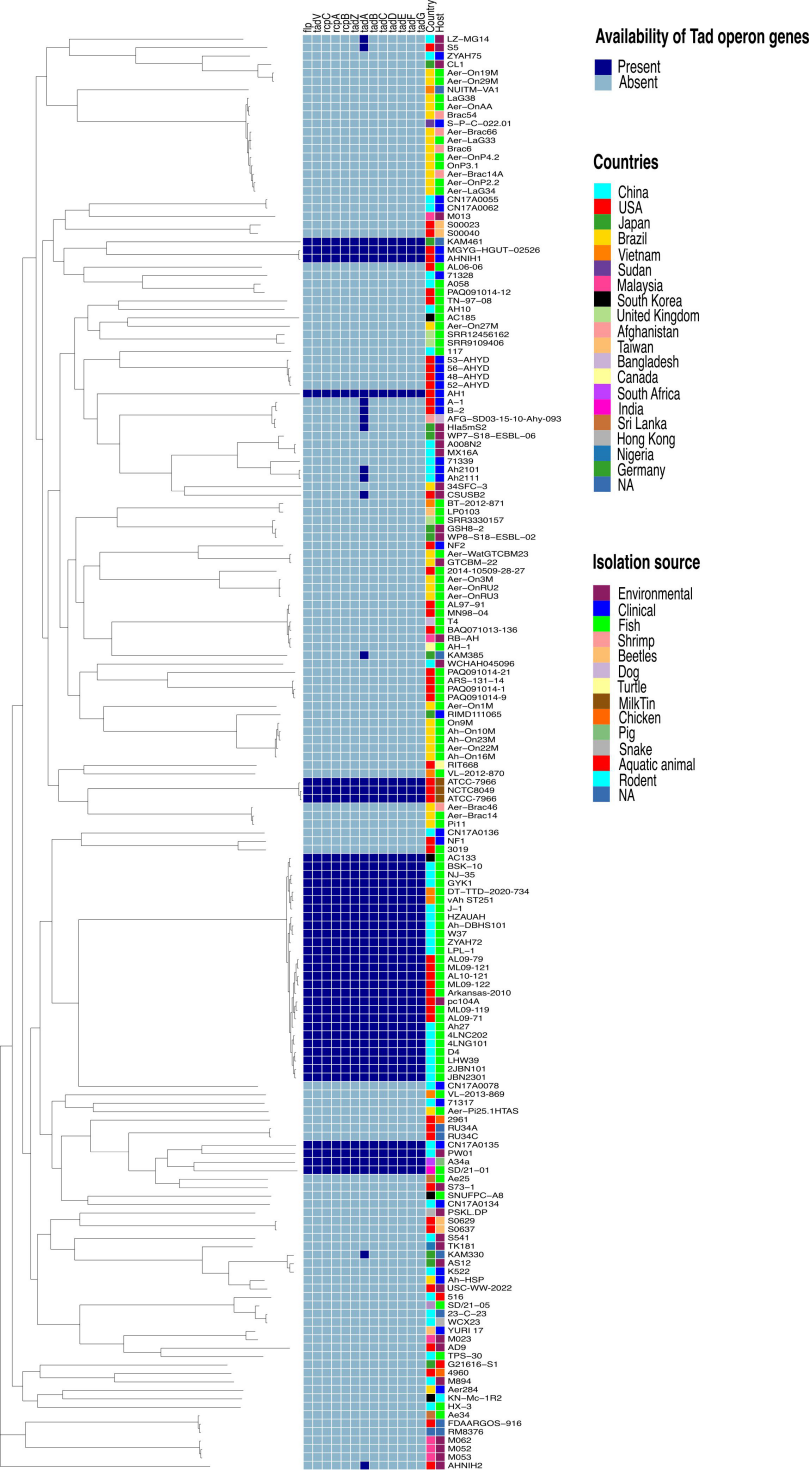
Phylogenetic tree based on the *Aeromonas hydrophila* core genome and availability of individual Tad operon genes across 170 *A. hydrophila* genomes. Isolation sources and geographical locations for each genome are included. Navy blue color represents the presence of Tad operon genes, whereas light blue represents absence. Branch lengths were reduced to fit the image.

The virulence factor database (VFDB) (Liu et al., 2022) and MacSyFinder tool were used with default features to identify individual Tad operon genes across the 170 *A. hydrophila* genomes investigated (Abby et al., 2014; Abby et al., 2024). Specifically, the “unordered” dataset option was chosen because most of the evaluated genomes were incomplete drafts. Briefly, the following parameters were used: the topology of the replicon was linear/circular, maximal E-value was 1.0, maximal independent E-value was 0.001, and the minimal profile coverage was 0.5. Both mandatory genes and accessory genes were identified. The protein sequences of the 13 Tad operon genes were identified using VFDB and MacSyfinder. Based on these findings, the 13 genes were organized in an operon structure (**Figure 1**). The 13 Tad operon gene protein sequences were then uploaded to CLC Genomics Workbench (version 23.0.3), where BLASTp searches were conducted to compare the 13 Tad genes against the 170 *A. hydrophila* genomes. Only matches with E-values 1×10^-20^ were considered present. The distribution of the predicted 13 Tad operon genes among 170 *A. hydrophila* genomes was presented using the pheatmap library in R Studio (Version 2023.06.0 + 421) (**Figure 2**). Also, the GC content of the Tad operon was analyzed and compared to the entire genome of *A. hydrophila* ML09-119 using SnapGene (version 6.2.1) (**Figure 1**).

### 2.3 Bacterial strains and plasmids

*A. hydrophila* strain ML09-119 (Tekedar et al., 2013) was used as a representative of the vAh clonal group impacting U.S. channel catfish aquaculture. The bacterial strains and plasmids used in this study are listed in **Table 2**. *Escherichia coli* strains CC118 λ*pir* and BW19851 were used for cloning and conjugation purposes, respectively, and suicide plasmid pMEG-375 was used to transfer gene mutations into *A. hydrophila*. Brain heart infusion (BHI) agar and broth (Difco, Sparks, MD, USA) were used to grow *A. hydrophila* strain at 30°C. Luria–Bertani (LB) agar and broth (Difco) were used for *E. coli* incubation at 37°C. When needed, the following antibiotics and reagents (Sigma-Aldrich, Saint Louis, MN, USA) were used in the given concentrations: colistin (12.5 μg/mL), chloramphenicol (10-25 μg/mL), ampicillin (100 µg/mL), mannitol (0.35%), and sucrose (5%).

**Table 2:** Bacterial strains and plasmids used in the present study.

Strain or plasmid	Description	Source
*A. hydrophila* ML09-119	Isolate from a disease outbreak on a commercial catfish farm.	(Griffin et al., 2013)
*vAh*Δ*tad*	*A. hydrophila* ML09-119 derivative; Δ*tad*	This study
*Escherichia coli*		
CC118λ*pir*	Δ(*ara*-*leu*); *araD*; Δ*lacX74*; *galE*; *galK*; *phoA20*; *thi-1*; *rpsE*; *rpoB*; *argE*(Am); *recAl*; λ*pir*R6K	(Herrero et al., 1990)
BW19851	*RP4-2 (Km::Tn7, Tc::Mu-1), DuidA3::pir+, recA1, endA1, thi-1, hsdR17, creC510*	(Metcalf et al., 1994)
Plasmid		
pMEG-375	8,142 bp, Amp^r^, Cm^r^, *lacZ*, R6K *ori*, *mob incP*, *sacR sacB*	(Dozois et al., 2003)
pAhΔ*tad*	10,173 bp, Δ*tad*, pMEG-375	This study

### 2.4 In-frame deletion of Tad operon in *A. hydrophila* ML09-119

In-frame deletion was used to create a complete Tad operon mutant in *A. hydrophila* ML09-119. Briefly, the Tad operon mutant was constructed by allelic exchange and homologous recombination using suicide plasmid pMEG-375 containing the counter selectable gene *sacB* (Dozois et al., 2003). Four primers (A, B, C, and D) were designed for the Tad operon region using Primer3 (https://primer3.ut.ee/) (Untergasser et al., 2012) (**Table 3**). Two specific compatible restriction enzyme sites were introduced in A and D primers for cloning, and the reverse complement of primer B was added to the 5′ end of primer C to create an overlap region to initiate the fusion of PCR fragments by overlap extension PCR (Horton et al., 1989). The upstream (AB fragment) and downstream (CD fragment) regions of the Tad operon were amplified using two sets of primers. Amplified PCR fragments AB and CD were annealed at the overlapping regions, and a single fragment was created using the A and D primers. The AD product was purified, digested, and ligated into digested pMEG-375, then transformed into *E. coli* CC118λpir, and the final product was grown on LB agar with ampicillin. The positive plasmids carrying the mutated Tad operon were transferred into *A. hydrophila* ML09-119 by conjugation using *E. coli* BW19851 strain. Transconjugants were selected on plates containing chloramphenicol and colistin. In this process, chloramphenicol was used to identify the integration of pMEG-375 in *A. hydrophila* ML09-119 chromosome, while colistin was used as counterselection against *E. coli*. For confirmation purposes, PCR was used to verify the vector had integrated correctly into the *A. hydrophila* ML09-119 chromosome. Following sucrose selection, colistin resistant and chloramphenicol sensitive colonies were selected, and the candidate mutants were screened by colony PCR using A and D primers. Mutant validation was done by sequencing AD fragments amplified from chloramphenicol sensitive mutants using Tad-ConF01 and Tad-ConR01 (**Table 3**). The final *A. hydrophila* Tad operon mutant was designated as *vAh*Δ*tad*.

**Table 3:** Primers used to generate and verify in-frame deletion of vAh Tad operon.

	Primer ID		Sequence 5-3’	RE
Tad	Tad-EF	A	AGCTC**ggatcc**ATAGTGCAACAGCCCTTCTTG	*BamHI-HF*
	Tad-IR	B	GTAATCCCAAACTCGCATGTC	
	Tad-IF	C	**GACATGCGAGTTTGGGATTAC** TAGT GCCATAGCGTGCAAGA	
	Tad-ER	D	AGCTC**tctaga**TCACCGACAAAATCCTCAATC	*XbaI*
	Tad-ConF01		GGTATCTCGCTGGAGCTGAC	
	Tad-ConR01		TGATGAGGGTGCTGTAGTGC	

Bold letters show restriction enzyme (RE) recognition sequences added to primers. Underlined bold letters indicate reverse complement of Tad-IR primer sequence.

### 2.5 Verification of Tad mutant, *vAh*Δ*tad*, by complete genome sequencing

The *vAh*Δ*tad* was expanded in 9 mL porcine brain heart infusion broth (Becton Dickinson, Franklin Lakes, NJ, USA) in static, overnight cultures at 28°C. An aliquot (3 mL) of the expanded culture was pelleted by centrifugation (20,000 x g). High molecular weight genomic DNA was isolated from the concentrated pellet using the PureGene DNA isolation kit (QIAGEN, Hilden, Germany). Long reads were produced on a GridION platform (Oxford Nanopore Technologies, Oxford, UK) using the rapid barcoding kit (RBK004) and v9.4.1 flow cells. These sequences were filtered to an average quality 10 using NanoFilt v2.2.0 (Bolger et al., 2014) to produce 211,519,148 bases from 30,912 reads of at least 1000 bp with an average read length of 6842 bp. The genome was assembled into a single contig with Canu v1.7 (Koren et al., 2017), the consensus sequence was produced using Medaka v0.11 (Oxford Nanopore Technologies, https://github.com/nanoporetech/medaka), and the contig was circularized manually.

### 2.6 Virulence of the *vAh*Δ*tad* mutant in catfish fingerlings

All fish experiments were conducted under a protocol approved by the Institutional Animal Care and Use Committee (IACUC) at Mississippi State University. Virulence of *vAh*Δ*tad* was compared to *A. hydrophila* ML09-119 wild-type strain (vAh-WT) by immersion route of exposure as described previously (Abdelhamed et al., 2016). Briefly, specific-pathogen-free (SPF) channel catfish fingerlings (13.54 ± 0.54 cm, 15.32 ± 1.98 g) were stocked into twelve 40-liter flow-through tanks (14 fish/tank) and acclimated for a week. Tanks were assigned randomly to three treatment groups: *vAh*Δ*tad*, vAh-WT, and BHI (sham infected). Each group included three replicate tanks. Fish were fed twice daily with a commercial catfish feed, and water temperature was maintained at 30°C (±2) throughout the experiments. On the challenge day, the water levels in each tank were decreased to 5 L, and 100 mL of overnight culture were added directly to each tank (1.9 × 10^9^ CFU/mL water). After three hours, the water flow was restored, and fish were maintained as usual. Negative control (sham-infected group) tanks were treated similarly, but exposed to 100 mL of sterile BHI broth. During the immersion process, the water was well aerated. Fish mortalities were recorded daily for 7 days, and cumulative percent mortality calculated for each group.

### 2.7 Growth kinetics

Growth kinetics of *vAh*Δ*tad* and vAh-WT in BHI broth were compared. Briefly, bacterial growth curves were generated by measuring the optical density at 600nm (OD_600_) on a multimode reader (BioTek Cytation 5) every half an hour for 72 h at 30°C. The growth assays were conducted in two independent experiments, each with 24 replicates for *vAh*Δ*tad* and vAh-WT strains.

### 2.8 Biofilm formation

Biofilm formation of the *vAh*Δ*tad* and vAh-WT strains were compared using a microtiter plate assay. Five different colonies of each strain were cultured to log-phase (OD_600_ = 0.6 ± 0.05) at 28°C in BHI broth and diluted to OD_600_ of 0.1 using freshly prepared media. A 100 µL of the diluted cultures were added into a 96-well plate and incubated at 28°C overnight. Sterile BHI media (100 µL) was added to the blank control wells. For quantitative analysis, 8 replicate wells were used for each treatment and the control group. After incubation, the bacteria were discarded by turning the plate over and shaking out the liquid, then gently submerging the plate in a tub of water. The washing step was repeated twice to remove any unbound bacteria and media before staining. To each well, 125 µL of crystal violet (CV) (0.1%) was added and the plate was incubated at room temperature for 15 min. Next, the microtiter plate was rinsed 3-4 times by submerging in sterile water as described above, followed by vigorous blotting on a stack of paper to remove any unbound bacteria and dye. The plate was air-dried and 125 µL of 95% ethanol was added to each well to solubilize the CV. The plate was incubated for 15 min and the solubilized CV was transferred to a new flat bottom microtiter plate and absorbance was measured at 595nm. The biofilm assay was conducted with 35 biological replicates for both *vAh*Δ*tad* and vAh-WT strains.

### 2.9 Motility

The *vAh*Δ*tad* and vAh-WT strains were cultured to log-phase (OD_600_ = 0.6 ± 0.05) at 28°C in BHI broth. Two microliters of bacterial culture were spotted on a 0.6% BHI semi-solid plate and incubated at 28°C. Replicate plates were used for statistical analysis. The diameters of bacterial growth were measured after 16 hours of incubation. Each motility assay was conducted with 7 independent biological replicates, with 3 technical replicates per biological replicate, for both the *vAh*Δ*tad* and vAh-WT strains.

### 2.10 Temperature stress

The ability of the *vAh*Δ*tad* and vAh-WT strains to withstand extreme temperatures was evaluated. Both strains were cultured to log phase (OD_600_ = 0.6 ± 0.05) at 28°C and then adjusted to an OD_600_ of 0.1 using sterile BHI broth. The bacterial suspension was subsequently incubated at 18°C, 28°C (control), and 37°C for 24h at 180 rpm. Following incubation, the cultures were washed twice with sterile phosphate-buffered saline (PBS) and spread on BHI agar plates for colony counting and statistical analysis. The temperature assay was conducted with 4 biological replicates for both *vAh*Δ*tad* and vAh-WT strains.

### 2.11 Acid-alkali stress

The *vAh*Δ*tad* and vAh-WT strains were cultured to log-phase (OD_600_ = 0.6 ± 0.05) at 28°C in BHI broth. Bacterial pellets were collected, washed twice with sterile PBS, and incubated in BHI at pH 5.0, pH 6.0, pH 7.0 (control), pH 9.0, and pH 10.0 for 30 minutes at 28°C. Viable bacteria were quantified by spreading on BHI agar plates after serially diluting. The two strains’ percent viability (CFUs/mL) was calculated and compared. The acid-alkali stress assay was conducted with 3 biological replicates for both *vAh*Δ*tad* and vAh-WT strains.

### 2.12 Osmotic stress

Under optimal growth conditions at 28°C, the *vAh*Δ*tad* and vAh-WT strains were cultured to log-phase with an optical density of 0.6 ± 0.05 at 600nm. Bacterial pellets were collected, washed with PBS, and resuspended in fresh BHI broth containing 0.5 M sodium chloride (NaCl), which was then adjusted to OD_600_ of 0.1. Subsequently, 200 μL of each bacterial suspension was dispensed into separate wells of a 96-well microplate. To the control wells, 200 μL of sterile BHI broth was added. Growth was monitored every 1 h for 16 h at 28°C in an automatic microplate reader using absorbance at 600nm. The osmotic stress assay was conducted with four biological replicates for both *vAh*Δ*tad* and vAh-WT strains.

### 2.13 Oxidative stress

To evaluate the viability of the strains against environmental oxidants, both *vAh*Δ*tad* and vAh-WT strains were cultured to log-phase (OD_600_ = 0.6 ± 0.05) at 28°C in BHI broth. Bacterial pellets were collected and washed twice with sterile PBS solution before treatment with 0.1M hydrogen peroxide (H_2_O_2_), using PBS as control. Bacteria were incubated for 20 min at 28°C, and the viable bacteria were quantified by spreading them onto BHI agar. The percentage viability of the two strains in the treatment group (H_2_O_2_) compared to the control group (PBS) was calculated and compared between the mutant and wild-type strain. The oxidative stress assay was conducted with four biological replicates for both *vAh*Δ*tad* and vAh-WT strains.

### 2.14 Scanning electron microscopy

The effects of Tad pili on the membrane structure of *A. hydrophila* was evaluated using scanning electron microscopy. Both the *vAh*Δ*tad* and vAh-WT strains were grown at 30°C. Pellets were collected from log phase and overnight cultures, and bacteria were fixed in 2.5% glutaraldehyde and 2% paraformaldehyde in 0.1M sodium cacodylate buffer for 2 hours at room temperature prior to fixation in 1% osmium tetroxide for one hour. Fixed pellets were then dehydrated in a graded ethanol series (10%, 20%, 30%, 50%, 70%, 80%, 85%, 95%, 100%), dried via a critical point dryer, mounted on aluminum stubs, and sputter coated with 30nm platinum. Images were captured using a JEOL 6500F field emission SEM operating at 10KeV at the Mississippi State University Institute for Imaging and Analytical Technologies.

### 2.15 Statistical analysis

Mean cumulative percent mortality data were arcsine transformed, and analysis of variance (ANOVA) was applied using PROC GLM in SAS for Windows v9.4 (SAS Institute, Inc., Cary, NC). For the characterization assays, the colony forming units/mL between the *vAh*Δ*tad* and vAh-WT strains were compared and analyzed using one-way analysis of variance (ANOVA). An alpha level of 0.05 was used in all analyses.

## 3 Results

### 3.1 Distribution of Tad operon in *A. hydrophila* isolates

This study comprehensively analyzed 170 *A. hydrophila* genome sequences derived from diverse geographical locations and hosts (**Table 1**), several of which originated from catfish disease cases in Alabama and Mississippi (Tekedar et al., 2013; Tekedar et al., 2015; Tekedar et al., 2016a; Tekedar et al., 2016b; Tekedar et al., 2017). Isolate identity was confirmed by ANI, and all selected genomes displayed ANI values of 95% or higher, indicating they are all the same species (Goris et al., 2007). Subsequently, a phylogenetic tree was constructed for the 170 *A. hydrophila* strains based on a core set of 1893 genes per genome, totaling 321,810 genes. The core genome contained 1,711,702 amino acid residues per base pair or 290,989,340 in total. Notably, the vAh isolates formed a distinct cluster with 100% branch conservation, and this separation was verified by ANI and average amino acid identity (AAI) values (data not shown here).

Furthermore, analysis of the *A. hydrophila* ML09-119 genome revealed a 10,852 bp operon containing 13 genes (*flp*, *tadV*, *rcpC*, *rcpA*, *rcpB*, *tadZ*, *tadA*, *tadB*, *tadC*, *tadD*, *tadE*, *tadF*, *tadG*) within the range of AHML_RS08095 to AHML_RS08155, collectively encoding the Tad operon (**Figure 1**). The GC content of the Tad operon was 64.82%, while the average GC ratio of the entire genome was 60.82% (**Figure 1**). *In silico* analysis of Tad operon distribution across the 170 *A. hydrophila* genomes confirmed the presence of the Tad operon in all U.S. and Chinese (24 genomes) vAh isolates (**Figure 2**). Additionally, human clinical isolates, particularly from the U.S. (strains AH1, MGYG-HGUT-02526, and AHNIH1) and China (strain CN17A0135) encoded Tad operon (**Figure 2**). Interestingly, most non-vAh isolates did not encode the Tad operon, with the exception of isolates PW01 from swimming pool water, A34a from swine, SD/21-01 from rohu carp (*Labeo rohita*), KAM461 (origin unknown), and NCTC8049 and ATCC 7966 from milk tins (**Figure 2**). Sequencing of the Tad mutant, *vAh*Δ*tad*, confirmed deletion of the Tad operon and the absence of additional random mutations outside the targeted locus.

### 3.2 Tad operon deletion impairs virulence and biofilm formation of *A. hydrophila* ML09-119

**Figure 3:**
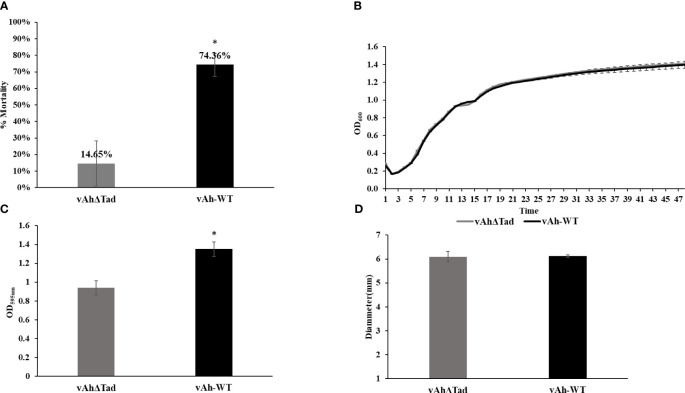
Evaluating the role of Tad operon in *A*. *hydrophila* ML09-119. The *vAhΔtad* and vAh-WT strains were compared and analyzed using one-way analysis of variance (ANOVA). An alpha level of 0.05 was used for all analyses, with asterisks (*) indicating statistical significance. **(A)** Percent mortalities of fish immersion challenge. The fish were exposed to 2 treatments, *A*. *hydrophila and vAh*Δ*tad.* Fish mortalities were recorded daily for 7 days, and percent mortalities was calculated for each treatment. **(B)** Growth Curve. Bacterial curves were generated by measuring the optical density at 600nm (OD_600_) on a multimode reader every half an hour for 72h at 30°C. **(C)** Biofilm assay. The strains were cultured to log phase and adjusted to OD_600_ of 0.1. The cultures were added to 96-well plate and incubated at 28°C for 24h. The cultures were washed and air-dried post staining with crystal violet (CV) for 15min at room temperature. The CV was solubilized with 95% ethanol for 15min, and absorbance was measured at 595nm. **(D)** Motility assay. The strains were cultured to log-phase. Two microliters of the culture were spotted on 0.6% BHI semi-solid plates and incubated at 28°C for 24h. The motility diameter for both strains were measured in millimeters.

To investigate the role of Tad pili in *A. hydroph*ila ML09-119 virulence, infectivity trials were conducted in catfish fingerlings. The *vAh*Δ*tad* strain exhibited a significantly lower mortality rate (14.65%) compared to the vAh-WT strain (74.36%) 72 hour post-infection (**Figure 3A**). Next, the impact of deletion of the Tad operon on growth kinetics of vAh isolate ML09-119 in BHI broth was assessed, revealing no measurable differences in growth (**Figure 3B**). Furthermore, the role of the Tad pili on biofilm formation was evaluated. The *vAh*Δ*tad* strain displayed significantly reduced (p ≤ 0.05) biofilm formation compared to the vAh-WT strain (**Figure 3C**), indicating that Tad pili are crucial for biofilm formation. No significant difference in motility was detected between the mutant and wild type parent (**Figure 3D**).

### 3.3 Role of Tad pili in stress resistance

**Figure 4:**
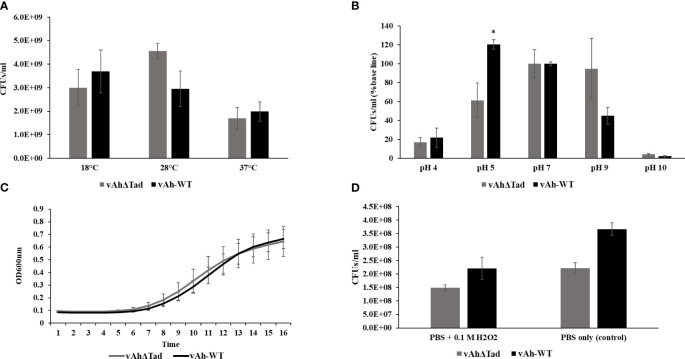
Characterization of v*Ah*Δ*tad* under different stress conditions. For the characterization assays, *vAhΔtad* and vAh-WT strains were compared and analyzed using one-way analysis of variance (ANOVA). An alpha level of 0.05 was used for all analyses, with asterisks (*) indicating statistical significance. **(A)** Temperature stress assay. The strains were cultured to log phase and OD_600_ was adjusted to 0.1 with BHI medium. The bacterial suspension was incubated at 18°C, 28°C (as a control) and 37°C for 24h. The cultures were double diluted with sterile PBS and plated onto BHI agar, for colony counting and statistical analysis. **(B)** Acid-Alkali tolerance. The strains were cultured at log-phase and the pellet was collected, washed twice with sterile PBS, and subjected to treatment in BHI at pH 4.0, pH 5.0, pH 7.0 (as a control), pH 9.0, and pH 10.0 for 30 minutes at 28°C. Viable cells were counted and compared for both strains by plating on BHI agar after serial dilutions. **(C)** Osmotic stress assay. The strains were cultured to log phase; pellets were collected and resuspended in BHI with 0.5M NaCl to adjust the OD_600_ of 0.1. The OD_600_ was monitored every 1h for 16h at 28°C. **(D)** Oxidative stress assay. The strains were cultured to log phase; pellets were collected and treated for 20min in PBS with 0.1M H_2_O_2_ and PBS without H_2_O_2_ as a control. Viable cells were counted on BHI plates after incubating for 24h.

The influence of Tad in the tolerance of *A. hydrophila* to environmental stressors, including temperature, pH, NaCl, and H_2_O_2_ was evaluated. The *vAh*Δ*tad* mutant strain exhibited growth to a higher density at 28°C than vAh-WT strain, but there were no observed differences in growth at 18°C and 37°C (**Figure 4A**). Under acid-alkali stress, both strains demonstrated similar growth at neutral pH 7.0, with a baseline percent viability of 100% (**Figure 4B**). However, as the pH deviated from neutrality, distinct trends emerged. Under acidic conditions (pH 5.0) the *vAh*Δ*tad* strain demonstrated significantly reduced viability compared to the vAh-WT strain (p ≤ 0.05) (**Figure 4B**). Under osmotic stress conditions, no significant difference was observed in the growth kinetics between the *vAh*Δ*tad* mutant and the vAh-WT strain. However, the *vAh*Δ*tad* strain had a shorter log-phase than the vAh-WT strain, suggesting a potential adaptation that renders it slightly quicker to adapt to osmotic stress (**Figure 4C**). Following exposure to hydrogen peroxide (H_2_O_2_), both the *vAh*Δ*tad* mutant and the vAh-WT strain exhibited decreased viability, with no observed differences between the mutant and the wild-type, indicating that Tad does not confer tolerance to oxidative stress in *A. hydrophila* (**Figure 4D**). Collectively, these results indicate an impact of Tad pili on acid tolerance in vAh strain ML09-119, but not temperature or osmotic stress.

### 3.4 Tad pili affect bacterial surface structure in *A. hydrophila* ML09-119

**Figure 5:**
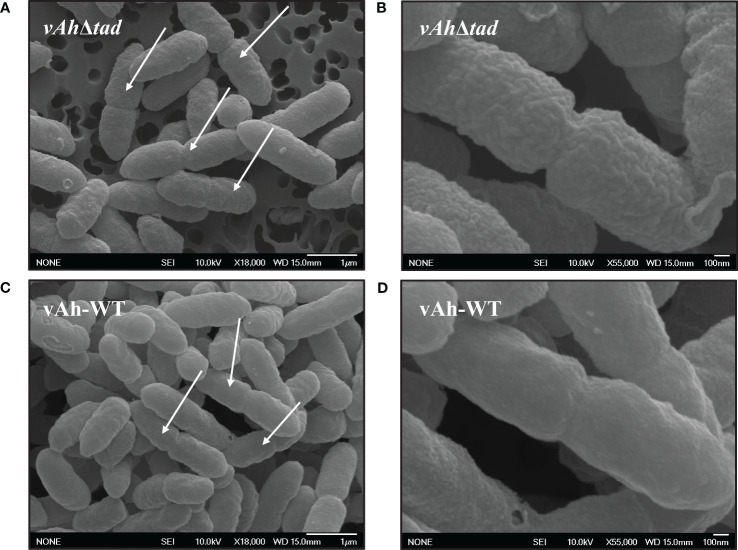
Ultrastructural observation of *vAh*Δ*tad* and vAh-WT. Bacterial strains were grown to log-phase in BHI at 30°C and specimens were examined with JEOL 6500F field emission SEM operating at 10 KeV. The white arrows indicate dividing cells. **(A)** Cell structure of *vAh*Δ*tad* at 18,000x and **(B)** at 55,000x under SEM. **(C)** Cell structure of vAh-WT at 18,000x and **(D)** at 55,000x under SEM.

Scanning electron microscopy was used to evaluate the effects of Tad pili on the surface structure of *A. hydrophila* ML09-119. While overnight cultures showed no apparent differences (data not shown), we observed changes in the bacterial surface morphology during the log-phase. In the log phase, vAh-WT strain displayed a distinctly smooth material covering its surface, a feature absent in the *vAh*Δ*tad*, which exhibited a surface that appeared rough (**Figures 5B, D**). In addition, the vAh-WT strain possessed prominent protrusions on its surface, while *vAh*Δ*tad* lacked these distinctive surface features (**Figures 5A, C**).

## 4 Discussion

Amidst the dynamics of host-pathogen interactions, pathogens are driven to persistently evolve, adapt to varied ecological niches, and evade the host’s immune defenses. These adaptations often occur by acquiring unique genes, toxins, and systems through horizontal gene transfer, gene fissions, or gene duplications, which leaves a distinct imprint on the pathogen’s genetic makeup. Comparative genomics allows identification of these unique genetic adaptations, which can be specific for a bacterial species or for a genetic lineage within a species. *A. hydrophila* is a genetically diverse species that has many unique lineages with varied environmental adaptations, virulence, and host specificities.

Based on previous work, vAh isolates consistently encode a unique T4cP, known as tight adherence (Tad), which is largely absent from non-vAh isolates (Tekedar et al., 2019). Based on this key finding, it was hypothesized that acquisition of the Tad operon is an adaptive advantage, potentially contributing to the pathogenicity of vAh isolates. To test this hypothesis, a comprehensive comparative genome analysis was conducted with 170 *A. hydrophila* genomes, sourced from diverse hosts and distinct geographical locations. All evaluated genomes shared an ANI ≥95%, indicating conspecificity (Kim et al., 2014). A core-genome based phylogenetic tree revealed that vAh isolates from different geographical origins form a discrete subgroup (Hossain et al., 2014; Tekedar et al., 2019). These analyses also support the notion that U.S. and Chinese vAh isolates originated from the same monophyletic clade. Furthermore, *in silico* analysis confirmed previous findings that vAh isolates consistently carry the Tad operon.

Homologs of Tad loci are widely distributed across plant, human, and animal pathogens such as *Sinorhizobium meliloti* (Zatakia et al., 2014), *Yersinia pestis* (Tomich et al., 2007)*, Bifidobacterium breve* (O'Connell Motherway et al., 2011; Ellison et al., 2019; O'Connell Motherway et al., 2019; Alessandri et al., 2021), *Vibrio vulnificus* (Pu et al., 2018a; Duong-Nu et al., 2019; Zhang et al., 2021)*, Myxococcus xanthus* (Seef et al., 2021), *Mycobacterium tuberculosis* (Hosseini et al., 2014; Ramsugit and Pillay, 2015; Mann et al., 2016; Alteri et al., 2022), *Caulobacter crescentus* (Skerker and Shapiro, 2000; Mignolet et al., 2018; Hershey et al., 2019; Sangermani et al., 2019; Mignolet et al., 2021), and *Cutibacterium acnes* (Davidsson et al., 2017). The presence of the Tad system in such diverse bacterial species hints at an adaptive advantage, substantiated by its diverse roles. Tad pili have been implicated in biofilm formation, which provides a protected environment that enables increased resistance to antibiotics, biocides, and immune defenses. Therefore, biofilms facilitate environmental persistence and chronic bacterial infections (Pu et al., 2018a; Duong-Nu et al., 2019). Additionally, Tad pili are associated with enhanced resistance to serum bactericidal activity, a critical factor in bacterial survival within the host (Duong-Nu et al., 2019). What distinguishes Tad from other Type IV Pilus (T4P) systems is its versatility, encompassing participation in predator-prey interactions (Seef et al., 2021), invasion of host tissue (Duong-Nu et al., 2019), establishment of symbiotic relationships (Nykyri et al., 2013; Zatakia et al., 2014), modulation of cellular signaling within the host (Bernard et al., 2009; O'Connell Motherway et al., 2019), persistent infections in prostate tissue (Davidsson et al., 2017), and natural competence (Angelov et al., 2015; Cai et al., 2021). Furthermore, investigations into other bacterial species have highlighted the importance of the Tad pili in host invasion, survival, and resistance to complement activation (Duong-Nu et al., 2019).

In particular, Tad pili serve a unique function in the early stages of infection by acting as an adhesive bridge, facilitating the attachment of pathogens to host cells. For instance, Tad pili are indispensable for *Bifidobacterium breve* to colonize the human gut. In particular, the *tadE* gene contributes to host signaling that promotes colonic host epithelial proliferation (O'Connell Motherway et al., 2019). Similarly, in *S. meliloti*, the Tad locus is necessary for competitive bacterial-plant symbiotic interactions (Zatakia et al., 2014).

In vAh ML09-119, a set of 13 Tad genes organized within an operon structure were identified (**Figure 1**). The function of Tad in vAh was investigated through the generation of a knockout mutant that encompassed the entire operon. Compared to the wild-type strain, there was significantly reduced mortality in catfish fingerlings exposed to *vAh*Δ*tad* indicating the Tad operon plays a critical role in the virulence of *A. hydrophila* ML09-119 (**Figure 3A**). This finding is consistent with the role of the Tad operon in diverse host-pathogen systems.

In contrast to these findings, studies on *Aeromonas salmonicida* (Boyd et al., 2008) and the human pathogen *M. tuberculosis*, which primarily focused on disruptions in a subset of the Tad operon genes, revealed the Tad operon was not essential for virulence in those host-pathogen interactions (Mann et al., 2016). Comparably, the current study involved a complete deletion of the Tad operon, resulting in significant attenuation. These contrasting outcomes could be driven by species- or host-specific nuances during host-pathogen interactions. For example, deleting Tad genes in a human pathogen, *M. tuberculosis*, did not impact biofilm formation or virulence in a mouse model (Davidsson et al., 2017). In contrast, the Tad operon in *M. tuberculosis* was upregulated upon interaction with human epithelial cells (Alteri et al., 2022), suggesting that differences in host environment may affect the function of Tad pili. Interestingly, *Cutibacterium acnes* type II isolates carrying plasmids with four Tad genes from human prostate cancer specimens were speculated to trigger infection-induced prostate cancer (Davidsson et al., 2017). Herein, a complete Tad operon was identified from some clinical *A. hydrophila* isolates from humans in the USA and China (**Figure 2**), suggesting Tad may facilitate *A. hydrophila* invasion of human cells. However, despite these observations, the precise role of Tad pili in *A. hydrophila* isolated from human cases remains a subject for future investigation.

A prominent hypothesis in studies focused on pathogens including *A. actinomycetemcomitans, Haemophilus ducreyi*, *Pasteurella multocida*, *V. vulnificus*, *C. crescentus*, *M. tuberculosis* and *S. meliloti*, suggests the Tad system has been horizontally transferred (Planet et al., 2003; Zatakia et al., 2014; Ramsugit and Pillay, 2015; Pu et al., 2018b; Beeby, 2019; Ellison et al., 2019). Evolutionary analyses suggest this genetic transfer coincided with the development of a ‘EppA-dependent’ (Epd) pilus homologue, acquiring a secretin, tracing their origins to an ancient genetic exchange with the Archaea (Denise et al., 2019; Sonani et al., 2023). It is thought the Tad operon may provide a competitive edge by facilitating niche adaptation, allowing the pathogen to persist in diverse environments.

Tad systems show considerable diversity among bacterial species, revealing distinct arrangements and functional roles. For instance, *M. tuberculosis* maintains only five Tad genes, diverging from the typical functional Tad loci which consists of 12-14 genes organized in a single operon. Other organisms like *Burkholderia pseudomallei*, *Mesorhizobium loti*, and *V. vulnificus* harbor three Tad loci (Hossain et al., 2014). In *V. vulnificus*, the deletion of all three Tad loci was necessary to mitigate pathogenicity (O'Connell Motherway et al., 2011). *S. meliloti*, on the other hand, encodes two Tad loci, situated in the chromosome and a megaplasmid (Kim et al., 2014). In comparison, *C. crescentus* has seven genes homologous to the Flp pilus genes; in this species, Flp pili serve as surface sensors altering secondary molecules controlling cell cycle events (Ellison et al., 2017; Sangermani et al., 2019). In *Mycobacterium luteus*, two Tad clusters located 1.2 Mbp apart are crucial for natural competence (Angelov et al., 2015).

In *A. hydrophila* ML09-119, the Tad operon has higher GC content (64.82%) than the average for the rest of the genome (60.82%) (**Figure 1**), suggesting acquisition from horizontal transfer. Interestingly, vAh isolates consistently encode the Tad operon while exhibiting the absence of any type III secretion system (T3SS) (data not shown here). This suggests the mechanism of pathogenesis for vAh, particularly how vAh manipulates function of host cells, is quite different from pathogens that utilize T3SS to inject effector proteins directly into host cells, and Tad may be an important adaptation in the absence of T3SS.

In addition to its role in pathogenicity, the effects of the Tad operon knockout on critical factors such as growth kinetics, motility, and biofilm formation in *A. hydrophila* was also assessed. Although deletion of the Tad operon had no effect on growth rate and motility, it significantly reduced biofilm formation. Tad pili have been reported as a crucial adhesion factors for initial surface attachment, a vital step in microcolony formation. They facilitate cell-cell interactions and aggregation, promoting the development and maturation of structured biofilms. Biofilms shield pathogens from environmental stressors and host defenses, enhancing survival against antimicrobial peptides and phagocytosis. Biofilms are a characteristic feature of chronic infections in numerous pathogens, including *Y. pestis* (causing bubonic plague) (Cui et al., 2020), *Vibrio cholerae* (responsible for gastrointestinal infections) (Huang et al., 2023), *M. tuberculosis* (Alteri et al., 2022), and *Bordetella pertussis* (causing whooping cough) (Tomich et al., 2007; Serra et al., 2011). Notably, in *V. vulnificus*, Tad pili mediate biofilms, which can facilitate resistance to serum killing (Duong-Nu et al., 2019). Notably, *A. hydrophila* isolated from biofilms results in chronic necrosis within 24 hours when injected into channel catfish muscle. Comparably, planktonic *A. hydrophila* failed to produce similar lesions, even up to seven days post-injection (Stewart and Franklin, 2008; Barger et al., 2021).

The significance of T4P extends beyond their conventional roles in attachment and motility, including the capacity to perceive cues within the environment and orchestrate responses. In the current study, Tad pili did not contribute to *A. hydrophila* resistance against most stress factors investigated (**Figure 4**). However, a significant contribution to survival at pH 5 was observed. Interestingly, in the human pathogen *M. tuberculosis*, acidic pH of the phagosomal vacuole was shown to trigger assembly of the Tad pilin (Alteri, 2005). In *A. hydrophila*, Tad pili may function as a sensor regulator for low pH, inducing expression of other adaptive mechanisms, or it may directly mediate resistance to low pH.

Structural observations under SEM revealed distinct characteristics in actively dividing *A. hydrophila*. vAh-WT exhibited a smooth layer absent in *vAh*Δ*tad* bacteria, which displayed rough, irregular protrusions with a coarse texture (**Figure 5**). Interestingly, these distinct surface modifications were only observed in the log-phase of growth and were absent during the stationary phase. Because Tad pili are a surface structure, it is not surprising that surface alterations were detected by SEM, but the significance of the rough surface during log phase is not currently known. In *C. crescentus*, Tad pili regulate secondary signaling pathways through mechanical sensing of surface cues, leading to the production of highly efficient progeny equipped for sustained attachment (Sangermani et al., 2019; Snyder et al., 2020). It is possible the bacterial surface alterations in *vAh*Δ*tad* impacted host cell adhesion and/or induction of virulence mechanisms through signaling pathways resulting in reduced virulence, but further research is needed.

Collectively, these investigations into the function of Tad pili in vAh isolate *A. hydrophila* ML09-119 provides valuable insights into its role in virulence and environmental adaptation. Comparative genomic analysis confirmed the consistent presence of the Tad operon in the vAh subclade of *A. hydrophila*, suggesting Tad is essential for vAh virulence in catfish, biofilm formation and resistance to acid stress. This work highlights the multifaceted role of Tad pili in *A. hydrophila* pathogenicity and physiology. The presence of the Tad operon in some human *A. hydrophila* isolates suggests it may contribute to pathogenesis in humans as well, but further investigation is needed. Regardless, these findings demonstrate a clear role of Tad pili in contributing to virulence of vAh in catfish, which is one of the most important diseases affecting catfish aquaculture in the southeastern United States. Further investigation is warranted to delineate the role of Tad pili in vAh pathogenesis and explore its potential for treatment and prevention of motile Aeromonas septicemia.

## Data availability statement

The original contributions presented in the study are included in the article/**Supplementary Material**, Further inquiries can be directed to the corresponding author.

## Ethics statement

The animal study was approved by Institutional Animal Care and Use Committee of the Mississippi State University. The study was conducted in accordance with the local legislation and institutional requirements.

## Author contributions

HT: Conceptualization, Data curation, Formal analysis, Funding acquisition, Investigation, Methodology, Project administration, Resources, Software, Supervision, Validation, Visualization, Writing – original draft, Writing – review & editing. FP: Data curation, Formal analysis, Investigation, Methodology, Validation, Visualization, Writing – original draft, Writing – review & editing. JB: Data curation, Resources, Software, Visualization, Writing – review & editing. MG: Data curation, Resources, Writing – review & editing. GW: Data curation, Resources, Writing – review & editing. SK: Conceptualization, Data curation, Methodology, Writing – review & editing. HA: Data curation, Methodology, Writing – review & editing. VD: Data curation, Formal analysis, Investigation, Methodology, Writing – review & editing. LH: Formal analysis, Methodology, Resources, Writing – review & editing. ML: Conceptualization, Data curation, Funding acquisition, Investigation, Methodology, Resources, Supervision, Writing – review & editing.
