# Supplementary material for: Tad pili contribute to the virulence and biofilm formation of virulent Aeromonas hydrophila
Source: Front Cell Infect Microbiol. 2024 Jul 31;14:1425624. doi: 10.3389/fcimb.2024.1425624 (PMC11322086; doi:10.3389/fcimb.2024.1425624)
Supplement: Supplementary file 1 [file Table_1.docx]

**Table 1.** Protein Identification and Locus Tags for Tad Operon Genes in *A. hydrophila* ML09-119.

|  | **Tad operon genes** | **Gene name** | **NCBI Annotation** | **Locus tag** | **protein ID** |
| --- | --- | --- | --- | --- | --- |
| 1 | *flp* | N/A | Flp family type IVb pilin | AHML_RS08095 | WP_011705350.1 |
| 2 | *tadV* | N/A | leader peptidase PilD | AHML_RS08100 | WP_016350147.1 |
| 3 | *rcpC* | *cpaB* | Flp pilus assembly protein CpaB | AHML_RS08105 | WP_016350148.1 |
| 4 | *rcpA* | *flpC* | type 4 pilus assembly secretin FlpC | AHML_RS08110 | WP_043118798.1 |
| 5 | *rcpB* | *flpD* | Flp fimbrial biogenesis protein FlpD | AHML_RS08115 | WP_016350150.1 |
| 6 | *tadZ* | N/A | P-loop NTPase | AHML_RS08120 | WP_016350151.1 |
| 7 | *tadA* | N/A | CpaF family protein | AHML_RS08125 | WP_016350152.1 |
| 8 | *tadB* | *flpG* | Flp fimbrial biogenesis protein FlpG | AHML_RS08130 | WP_016350153.1 |
| 9 | *tadC* | *flpH* | Flp fimbrial biogenesis protein FlpH | AHML_RS08135 | WP_043118801.1 |
| 10 | *tadD* | *flpI* | Flp fimbrial biogenesis protein FlpI | AHML_RS08140 | WP_016350155.1 |
| 11 | *tadE* | *flpJ* | Flp fimbrial biogenesis protein FlpJ | AHML_RS08145 | WP_016350156.1 |
| 12 | *tadF* | *flpK* | type 4 pilus assembly pseudopilin FlpK | AHML_RS08150 | WP_016350157.1 |
| 13 | *tadG* | *flpL* | Flp fimbrial protein FlpL | AHML_RS08155 | WP_016350158.1 |
|  |  |  |  |  |  |
